# Sustainable synthesis of silver nanoparticles from *Azadirachta indica*: antimicrobial, antioxidant and *in silico* analysis for periodontal treatment

**DOI:** 10.3389/fchem.2024.1489253

**Published:** 2024-10-15

**Authors:** Binapani Barik, Bhabani Sankar Satapathy, Gurudutta Pattnaik, Desai Vijay Bhavrao, Krishna Prasad Shetty

**Affiliations:** ^1^ School of Pharmacy and Life Sciences, Centurion University of Technology and Management, Bhubaneswar, Odisha, India; ^2^ GITAM School of Pharmacy, GITAM Deemed to be University, Hyderabad Campus, Hyderabad, Telangana, India; ^3^ Department of Clinical Science, College of Dentistry, Centre of Medical and Bio-Allied Health Science Research, Ajman University, Ajman, United Arab Emirates

**Keywords:** silver nanoparticles, *Azadirachta indica*, antimicrobial, antioxidant, molecular docking, ADMET

## Abstract

**Introduction:**

This study explores potential application of silver nanoparticles (AgNPs) to treat periodontal infection using *Azadirachta indica* leaf extract. The eco-friendly green synthesis process uses *Azadirachta indica* as a natural stabilizer and reducer, allowing AgNPs to be formed.

**Methods:**

Experimental AgNPs were characterized through transmission electron microscopy (TEM), Fourier-transform infrared spectroscopy (FTIR), Zeta potential, ultraviolet-visible spectroscopy (UV-Vis) etc. The antimicrobial, antioxidant potential of AgNPs was tested to identify its efficacy against periodontal infections.

**Results and discussion:**

AgNPs were found spherical, nanosized (86 nm), with negative surface charge (−26.9 mV). TEM study depicted clear formation of discrete nanosize particles with smooth surface texture. Results showed strong antibacterial and anti-oxidant action of experimental AgNPs, preventing biofilm growth and bacterial viability. A higher binding affinity was observed between Quercetin and the selected protein, which is implicated in bacterial growth and biofilm formation on teeth. The study suggests that *Azadirachta indica* derived AgNPs could be a safe, efficacious, and eco-friendly alternative in place of conventional therapies to treat periodontal infection. Future *in vivo* studies are however warranted.

## 1 Introduction

Periodontal disease is a global issue affecting millions, with 10%–15% of adults experiencing severe periodontitis. It causes tooth loss, pain, and reduced quality of life and is linked to health disorders (Gidiagba et al., 2023). The financial impact is substantial, with medical treatment, tooth extraction, and reduced work efficiency (Osherov et al., 2023). Periodontal disease is primarily caused by the buildup of dental plaque, a viscous biofilm of bacteria on teeth and gumline (Shareef et al., 2019). Key pathogens include diverse range of parasites including *Treponema denticola, Prevotella intermedia, Fusobacterium nucleatum*, *Eikenella corrodens*, *Porphyromonas gingivalis*, causing inflammation and tissue damage (Malandrakis et al., 2022; Ozdal and Gurkok, 2022). Modern periodontal treatments have made significant progress in managing periodontal disease, but they still face challenges such as insufficient effectiveness in advanced cases, limited access to care, patient adherence, and the possibility of disease return (Hossain et al., 2024; Khan et al., 2022). The increasing incidence of dental implants has led to concerns about peri-implantitis, a disorder that resembles periodontitis but affects dental implants (Xu et al., 2022; Grizzo et al., 2023). Antibiotic resistance has also been wide spread incidence owing to irrational use of antibiotics in periodontal infections (Harish et al., 2023; Kumar et al., 2023).

*Azadirachta indica*, a plant largely popular for its unique flavonoids content and long history of use in traditional medicine system, has also been found to be effective in treating periodontal disease (Hu et al., 2023). Flavonoids content of the *Azadirachta indica* leaf extract have antibacterial, antifungal, and anti-inflammatory properties, preventing the development of infections and reducing inflammation (Bajpai et al., 2022; Singh and Mijakovic, 2022a). They also possess antioxidant properties, protecting gum tissues from oxidative stress. *Azadirachta indica* extracts can also enhance tissue regeneration and wound healing and can be used in oral hygiene practices (Gupta et al., 2023).

Nanoparticulate mediated delivery has been emerged as hopeful technologies in improving pharmacological effectiveness and also in modulating pharmacokinetic and pharmacodynamic properties of conventional therapeutics (Vijayaram et al., 2024). Silver nanoparticles (AgNPs) are being explored as a potential treatment for periodontal disease thanks to their strong antibacterial properties and ability to infiltrate bacterial biofilms. AgNPs have been found to reduce inflammatory responses, improve periodontal tissue regeneration (Habeeb Rahuman et al., 2022). Few reports have documented effective antimicrobial potency of AgNPs against periodontal infections (Halilu et al., 2023). A recent study by Hedayatipanah et al. (2024), reported green synthesis of AgNPs from propolis and its antimicrobial activity on the on the *Porphyromonas gingivalis* biofilm. Similarly, in another study, AgNPs conjugated with chlorhexidine or metronidazole demonstrated preferential antibacterial and anti-inflammatory potency *in vitro* (Ortega et al., 2022).

The work aims to explore the use of *Azadirachta indica* leaf extract as a reducing and stabilizing agent for producing AgNPs for periodontal therapy. Primary objective was to create a reliable method for generating AgNPs with controlled dimensions, morphology, and surface properties. To achieve the highest yield, stability, and antibacterial activity, the synthesis parameters were optimized. Experimental AgNPs were then examined using techniques like UV-Vis spectroscopy, FTIR, size analysis, zeta potential and TEM (Huq et al., 2022). The antimicrobial activity of AgNPs against common periodontal pathogens was assessed using standard microbiological tests and antioxidant potential was evaluated using DPPH assay (Huq et al., 2022). Additionally, docking and ADME analysis was performed to unveil the mechanism of interaction with key periodontal bacterial protein.

## 2 Materials and methods

### 2.1 Materials

The green leaves of *Azardircata indica* were gathered from the centurion university campus, odisha. The silver nitrate used in this study was acquired from Mahavir Chemical Supply, located in Bhubaneswar, Odisha.

Nutrient agar media, nutrient broth, and antibiotic assay media serve as standard substrates for antimicrobial studies. The free radical scavenging activity of the nanoparticles was assessed using the DPPH assay, employing methanol or ethanol as solvents and ascorbic acid obtained from Himedia, Mumbai, India. All the chemicals were of analytical grade.

### 2.2 Methods

#### 2.2.1 Preparation of *Azadirachta indica* extract

The process of preparing an aqueous extract from *Azadirachta indica* leaves involved collecting the leaves and allowing them to dry completely in the shade. Once dried, the leaves were powdered and stored in an airtight container (Prakash et al., 2022; Adaramola et al., 2023). To make the extract, 5 gm of *Azadirachta indica* powder was boiled with 50 mL of distilled water for 30 min in a beaker. After cooling it to room temperature, the liquid extract was filtered and was stored at a temperature for future use.

#### 2.2.2 Green synthesis of experimental silver nanoparticles

Green synthesis of AgNPs was carried out as per the method reported elsewhere. Briefly, a solution of silver nitrate was prepared by dissolving 10 mg of silver nitrate in 50 mL of double distilled water. The solution was heated to a temperature range of 50°C–60°C, and *Azadirachta indica* extract was slowly added. The solution was thereafter incubated at a temperature of 80°C for duration of 60 min until an identifiable change in color was seen. The presence of pale-yellow color in the solution suggested the creation of AgNps (Hameed et al., 2022; Dubey and Mittal, 2020). This was further confirmed by the visible absorbance peak at 438 nm.

#### 2.2.3 Characterization of experimental silver nanoparticles

##### 2.2.3.1 UV–visible spectroscopy

The UV-visible absorption spectra were used to determine the optimal time and temperature conditions for the reduction of silver ions by the colloidal mixture of prepared AgNPs and the substrate mixture of the plant extract. This spectroscopic technique allowed for precise control over the synthesis process, ensuring maximum efficiency in the production of silver nanoparticles (Wade, 2021; Sedghi et al., 2021).

##### 2.2.3.2 Zetapotential and particle size

The zetasizer equipment was used to assess the particle size range and polydispersity of the nanoparticles. The particle size was determined by analyzing the temporal fluctuations in the scattering of laser light while the particles were undergoing Brownian motion (Dutt et al., 2023; Chinnasamy et al., 2021). This tool allows for the analysis of the average size of the particles in the sample. Overall, these analyses provide valuable insights into the characteristics and behavior of the nanoparticles in the colloidal solution (Nesappan and Subramani, 2023).

##### 2.2.3.3 Fourier transmission infrared spectroscopy (FTIR)

The FTIR spectroscopy was used to identify the functional groups and their interactions in the sample. The resultant peaks corresponding to specific chemical bonds provide valuable information about the composition of the reaction mixture**.** The resultant AgNPs was scanned over a wave length of 4,000 cm^−1^ to 600 cm^−1^ in an FTIR instrument (SHIMADZU IR Prestige-21, Mumbai, India).

##### 2.2.3.4 Morphological examination

The morphological examination allowed for the observation of the size, shape, and surface features of the AgNPs using the Transmission electron microscopy (TEM) and Selected Area electron diffraction (SAED). The analysis provides high-resolution images of the AgNPs, allowing for a detailed examination of their surface morphology (Ijaz et al., 2022; Dashora et al., 2022). Additionally, SAED analysis helps to determine the crystalline nature of the nanoparticles by analyzing the diffraction patterns produced when electrons interact with the sample (Mallineni et al., 2023).

##### 2.2.3.5 SAED analysis

The Tecnai G2 20 S-TWIN instrument was utilized to conduct measurements of selected area electron diffraction pattern. These measurements allowed for a detailed analysis of the crystal structure and orientation of the silver nanoparticles. This information is crucial in understanding the physical properties of the AgNPs (Hameed et al., 2022).

##### 2.2.3.6 TEM analysis

TEM imaging allows for direct observation of the nanoparticles, enabling precise determination of their size and morphology. It is a powerful imaging technique that allows for high-resolution visualization of nanoscale structures, such as AgNPs (Amananti et al., 2022). TEM provides detailed information about the size, shape, and distribution of nanoparticles within a sample (Sharma et al., 2022). The specimens were prepared by applying a droplet of suspension with an approximate thickness of 60 nm onto a carbon membrane. The carbon membrane was then transferred onto a copper grid and allowed to dry before being inserted into the TEM (Hashemi et al., 2022).

##### 2.2.3.7 Antimicrobial activity

*Treponema denticola* and *Porphyromonas gingivalis*, commonest pathogens associated with periodontal diseases are selected for the study (Hasan et al., 2022). These are known to be highly resistant to conventional antibiotics (Liaqat et al., 2022). Briefly, the agar plates were inoculated with the bacterial strains of *Staphylococcus. aureus* (ATCC 25923) *P. gingivalis* (ATCC 33277), *T. denticola* (ATCC 35405) and incubated at an optimal temperature for growth (Gonfa et al., 2023). A chlorhexidine suspension at an equivalent concentration was introduced into another sterile disc as a standard. The growth inhibition zones around each disc were measured after incubation. The nutrient broth medium provided essential nutrients for the bacteria to grow and multiply, allowing for an accurate assessment of their susceptibility to the AgNPs (Moges and Goud, 2022). 1 mg/mL solution of AgNPs was prepared using Milli Q water and subjected to appropriate sonication. Overnight grown culture in Luria-Bertani broth of the mentioned microorganisms was taken and diluted to an optical density of 1. A 500 μL volume of diluted bacterial suspension was evenly distributed across three distinct Luria-Bertani agar plates, each containing a different microorganism. Following the dispersion, two wells were created on each plate utilizing a well borer with an approximate diameter of 10 mm. Each of the three plates was filled with solutions of AgNPs at concentrations of 20 μg/mL, 50 μg/mL, and 75 μg/mL, and incubated for 24 h at 37°C. The antibacterial activity of AgNPs was assessed by measuring the presence of a clear zone of inhibition (ZOI) and from the minimum inhibitory concentration (MIC) (Chavan et al., 2023; Salem et al., 2022).

##### 2.2.3.8 Antioxidant study

The DPPH (2-Phenyl-1-Picrylhydrazyl) technique with relatively small modifications was used to assess the antioxidant activity of the biosynthesized AgNPs. DPPH provides free radical with a prominent purple color which gets decolorized in presence of antioxidant. This approach is a dependable way to assess the radical scavenging activity of a molecule (Owaid et al., 2021; Imchen et al., 2022).

Briefly, five distinct concentrations of biosynthesized AgNPs were prepared, with concentrations ranging from 0.5, 1, 1.5, 2, and 2.5 mg/mL. Each concentration was kept in a separate volumetric flask to which about 3 mL of a 0.1 mM methanolic solution containing DPPH radical was added. The solution was then rapidly agitated and left undisturbed for 30 min in a dark place at ambient temperature. The control sample included all the reaction reagents except for the AgNPs. Methanol was used for the purpose of baseline correction. Subsequently, the spectrophotometer was used to measure the absorbance at a wavelength of 517 nm. The findings were quantified as the percentage of radical scavenging activity, with ascorbic acid as a standard antioxidant.

The antioxidant activity data were quantified and reported as IC_50_values produced by linear regression analysis which is the minimum concentration (in micrograms) of the test sample needed to neutralize or block 50% of the radicals of DPPH concentration. Lower the IC_50_ value, the higher the antioxidant activity of the test sample.

The DPPH scavenging activity, expressed as a percentage of inhibition, is calculated using the formula:

$$\text{Antioxidant capacity }\left(\%\right)=\left\{\frac{\text{Abs}.\;\text{of}\;\text{control}-\text{Abs}.\;\text{of}\;\text{sample}}{\text{Abs}.\;\text{of}\;\text{control}}\right\}\times 100$$

##### 2.2.3.9 Molecular docking

To depict the molecular interaction in between quercetin with virulence factor of selected periodontal causing bacteria’s i.e., *P. gingivalis* and *T. denticola*, *in silico* docking analysis was employed. The molecular structure of the selected compound was constructed utilizing canonical smiles from the PubChem database PubChem CID: 5280343. The PDB structure of virulence factor of *P. gingivalis* and *T. denticola* was acquired from the RCSB Protein Data Bank (PDB id: 5Y1A and 3R15 respectively). Following that, the receptor and ligand structures were uploaded to the Argus Lab App for docking. The Argus Lab App outcome was modified for the optimal structure and visualized with Discovery studio visualizer 2.5. Subsequently, the relationship was verified by evaluation of the obtained binding energy, including ACE (atomic contact energy), global energy and attractive (vdw van der Waals). The proteins employed for the analysis included collagenase, protease, and urinase, which are responsible for catalyzing specific biochemical reactions in the oral cavity (Muraro et al., 2022; Satapathy et al., 2024).

##### 2.2.3.10 Assessment of ADMET

The Swiss ADME offers a practical alternative to the traditional approach of designing drugs from natural materials or synthetic compounds. The Swiss ADME approach was used to assess the pharmacokinetics, bioavailability, drug-likeness, and medicinal chemistry compatibility of the quercetin in order to enhance its properties and determining the bioavailability and efficacy of quercetin as a potential drug candidate.

## 3 Results and discussion

The main goal is to prepare nanomaterial-based therapies that are effective, non-hazardous, and compatible with the human body, potentially improving treatment outcomes and patient care in periodontal dentistry (Wasilewska et al., 2023; Singh and Mijakovic, 2022b).

In the current study, we have used *Azadirachta indica* leaf extracts to show the production of AgNPs. The impact of different experimental conditions on the biogenesis of silver nanoparticles was also investigated. In addition, silver nanoparticles that were produced under the most favorable circumstances were analyzed using several aspects of analytical equipment. The antibacterial and antioxidant activities of the produced silver nanoparticles were assessed. The results demonstrated that the silver nanoparticles exhibited strong antibacterial properties against a variety of pathogens. Furthermore, the antioxidant activity of the nanoparticles was found to be significant, indicating potential applications in biomedical and environmental fields (Kemala et al., 2022; Sharifi-Rad et al., 2024).

### 3.1 Green synthesis of silver nanoparticle from leaf extract of *Azadirachta indica*

**FIGURE 1:**
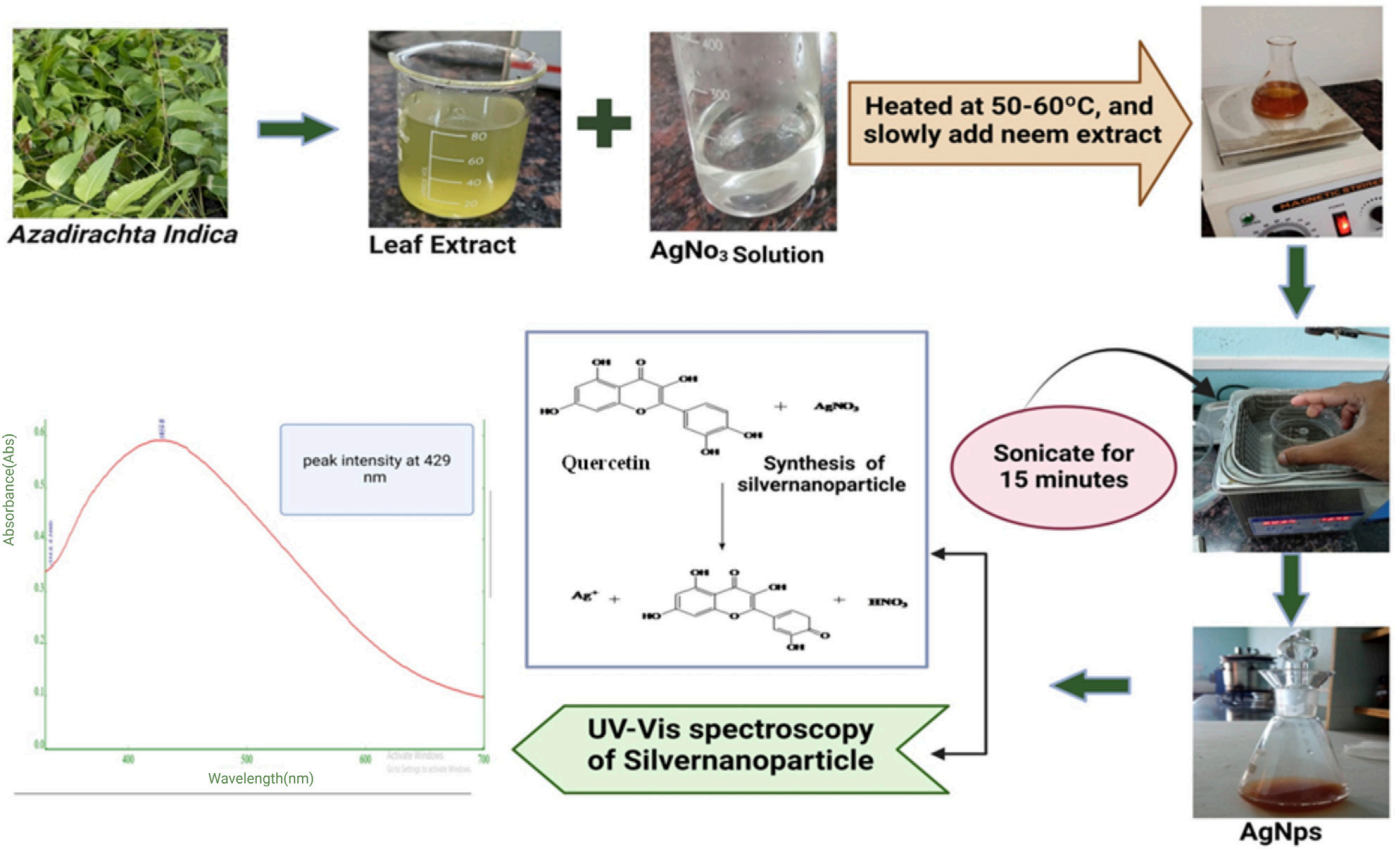
Outline of green synthesis steps of experimental Silver nanoparticles (AgNPs) from *Azadirachta indica* leaf extract.

The formation of silver nanoparticles was confirmed by surface plasmon resonance activity with a peak of 429 nm which indicated the reduction of AgNO_3_ and the subsequent formation of silver nanoparticles were validated, all achieved without the use of any hazardous substances (Karan et al., 2024; Hernández-Venegas et al., 2023). It can be determined that this is an ecofriendly friendly technique commonly referred to as green synthesis of silver nanoparticles using the *Azadirachta indica* extract as shown in Figure 1.

### 3.2 Characterization of optimized silver-nanoparticle

The characterization includes UV analysis, zetapotential, FTIR, SAED, and TEM. These techniques are used to determine the size, shape, and stability of the silver nanoparticles. UV analysis is used to measure the absorption spectrum of the nanoparticles, while zeta potential measures the surface charge. FTIR is used to analyze the chemical composition of nanoparticles, and SAED and TEM are used to visualize the crystal structure and morphology of the nanoparticles. Overall, these characterization techniques provide a comprehensive understanding of the optimized silver nanoparticles (Yun et al., 2022; Zhang et al., 2022a).

### 3.3 UV-visible spectroscopy

**FIGURE 2:**
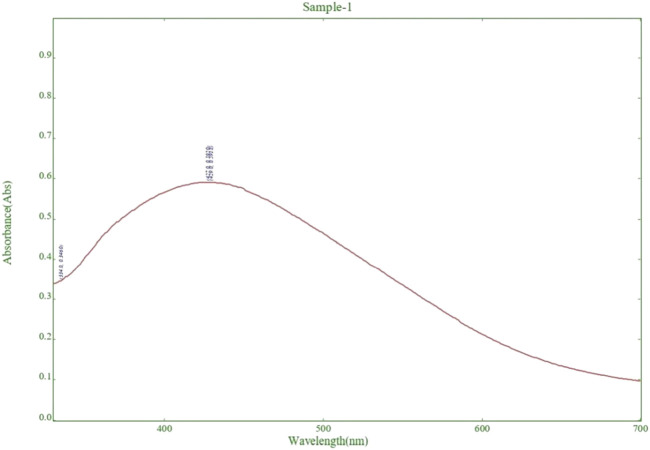
Detection of surface plasmon activity in experimental Silver nanoparticle (AgNPs) formation by UV-Visible spectroscopy.

The UV-vis absorption spectra showed a peak at 438 nm, indicating the formation of silver nanoparticles. As shown in Figure 2. The yellow color of the reaction mixture confirmed the successful reduction of Ag^+^ ions to AgNPs (Chavan et al., 2023). Analysis of the UV-vis spectra showed that the peak intensity at 438 nm increased with longer incubation time. This suggests that there were more AgNPs in the solution (Dutt et al., 2023). Additionally, the temperature had a significant effect on the reaction rate, with higher temperatures leading to faster synthesis of AgNPs. The stability and production of silver nanoparticles in water are tested using UV-Vis spectroscopy. Surface plasmon activity is detected during the formation of nanoparticles, as shown in Figure 2.

### 3.4 FTIR

**FIGURE 3:**
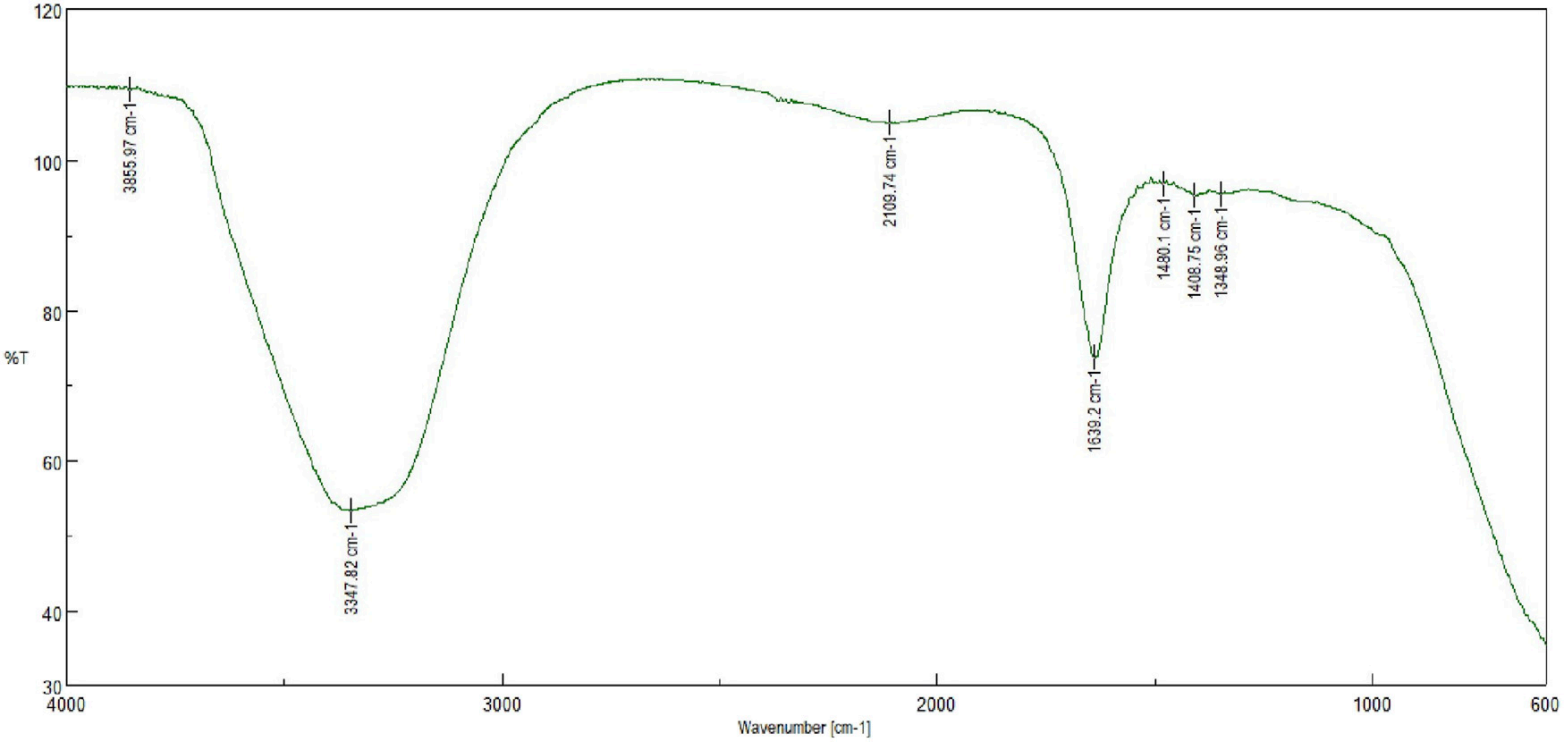
FTIR analysis of experimental Silver nanoparticles (AgNPs) showing characteristic peaks.

The FTIR value of AgNPS suggested that the nanoparticles have a strong absorption peak at around 400 cm^−1^, indicating the presence of silver-metal bonding. Additionally, the FTIR spectrum shows peaks at 1,348.96 cm^−1^ and 3,400 cm^−1^, suggesting the presence of carbonyl groups and hydroxyl groups on the surface of the nanoparticles. Overall, the FTIR analysis indicates that the AgNPS are well-formed and have functional groups that could potentially be useful for various applications in nanotechnology (Shirmohammadi et al., 2023; Nelagadarnahalli et al., 2023). The FTIR characterization image of AgNPs has been shown in Figure 3.

### 3.5 Zetapotential and particle size

**FIGURE 4:**
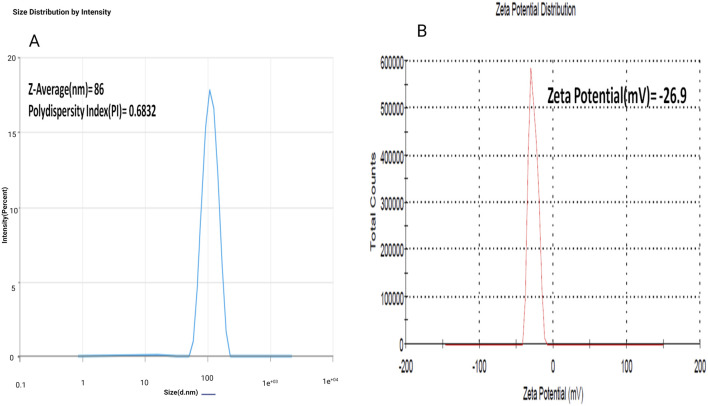
**(A)** Determination of avearge particle size (Z-avg), poly dispersity index (PDI) of experimental Silver nanoparticles (AgNPs); **(B)** Determination of zeta potential of experimental Silver nanoparticles (AgNPs).

The zeta potential of optimized AgNPs was found to be −26.9 mV, and the particle size was 86 nm with a polydisopersibility index of about 0.6832. This result suggests that the AgNPs would stable in solution due to their high negative zeta potential, indicating repulsion between particles. The relatively small particle size suggests good dispersion and the potential for enhanced reactivity in various applications. The low polydispersibility index also indicated a relatively uniform size distribution, further supporting the stability and potential effectiveness of the AgNPs (Abdel-Aty et al., 2023; Singh et al., 2020). As shown in Figures 4A, B.

### 3.6 SAED

**FIGURE 5:**
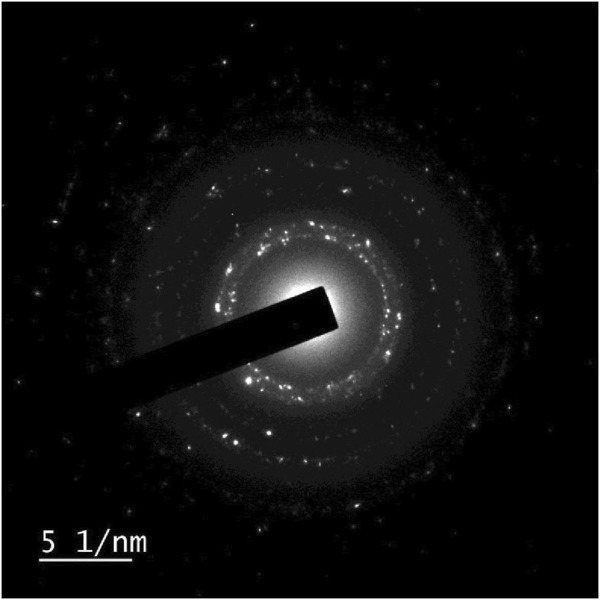
Selected area electron diffraction (SAED) characterization of experimental Silver. nanoparticles (AgNPs) to identify crystal structure and defects.

The results showed that the AgNPs had a uniform size distribution and exhibited a face-centered cubic [FCC] crystal structure. The Selected area electron diffraction (SAED) data obtained from the optimized AgNPs exhibits a circular ring that exhibits the crystalline characteristics of the silver nanoparticles. The dimensions and morphology of the nanoparticles play a crucial role, since their functionalities are contingent upon their size and form. This suggests that the optimized AgNPs were well-formed and possess high purity Figures 5.

### 3.7 TEM

**FIGURE 6:**
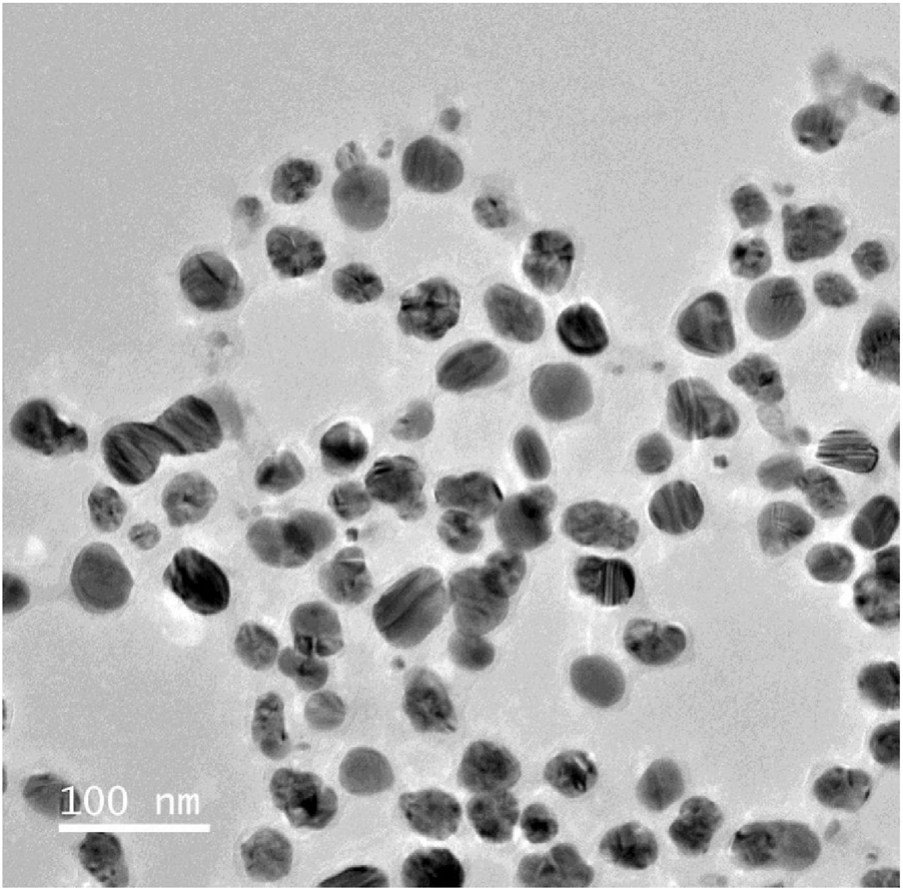
Transmision electron microscopic analysis of experimnetal Silver nanoparticles (AgNPs), showing uniform discrete nanosize particles.

This analysis determined the diameters ranging from 1 to 100 nm, revealed the existence of evenly distributed and highly crystalline silver nanoparticles. Most of them exhibited a roughly spherical morphology. The image showed that the periphery of the produced silver nanoparticles seemed to be less dense compared to the central region. Biomolecules function as capping agents, inhibiting the aggregation of nanoparticles (Ibrahim et al., 2021; Gontijo et al., 2020). As shown in Figure 6.

### 3.8 Antimicrobial activity

**FIGURE 7:**
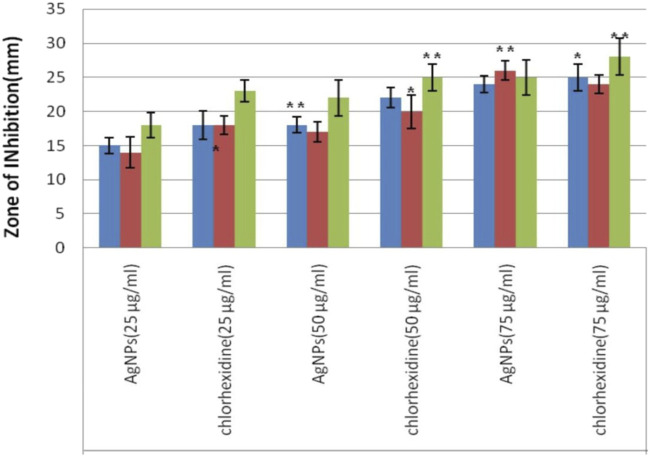
Evaluation of antimicrobial effect of experimental Silver nanoparticles (AgNPs) from measurement of zone of inhibition (ZOI) diameter against *S. aureus*, *T. denticola* and *P. gingivalis* at three different tested concentrations vs. Chlorhexidine as standard.

An antimicrobial study of silver nanoparticles showed promising results against a wide range of pathogenic bacteria and a standard drug to compare the effectiveness of AgNPs which shows a greater zone of inhibition against *P. gingivalish*, measuring 24 mm, *T. denticola*, measuring 22 mm; and *S. aureus*, measuring 20 mm, at a concentration of 75 μg/mL as shown in Table 1 (Donga and Chanda, 2021; Lomelí-Rosales et al., 2022). This research demonstrates that both Azadirachta indica and silver nanoparticles possess potent antibacterial abilities that successfully fight periodontal disorders (Yang et al., 2024; Zhang et al., 2022b). The large inhibition zone values showed that AgNPs could work well as an antibacterial agent against bacteria as shown in Figure 7.

**TABLE 1:** *In vitro* antimicrobial efficacy evaluation of AgNPs on selected periodontal pathogens, viz.*, S. aureus*, *T. denticola* and *P. gingivalis* through zone of inhibition assay.

Name of organism	Diameter of zone of inhibition [mm]					
	AgNPs (25 μg/mL)	Chlorhexidine (25 μg/mL)	AgNPs (50 μg/mL)	Chlorhexidine (50 μg/mL)	AgNPs (75 μg/mL)	Chlorhexidine (75 μg/mL)
*S. aureus*	15 ± 1.125	18 ± 2.052	18 ± 1.154	22 ± 1.456	24 ± 1.259	25 ± 1.961
*T. denticola*	14 ± 2.28	18 ± 1.32	17 ± 1.46	20 ± 2.45	26 ± 1.38	24 ± 1.34
*P. gingivalis*	18 ± 1.81	23 ± 1.61	22 ± 2.63	25 ± 1.95	25 ± 2.58	28 ± 2.67

Data are expressed Mean ± SD.

### 3.9 Antioxidant activity

**FIGURE 8:**
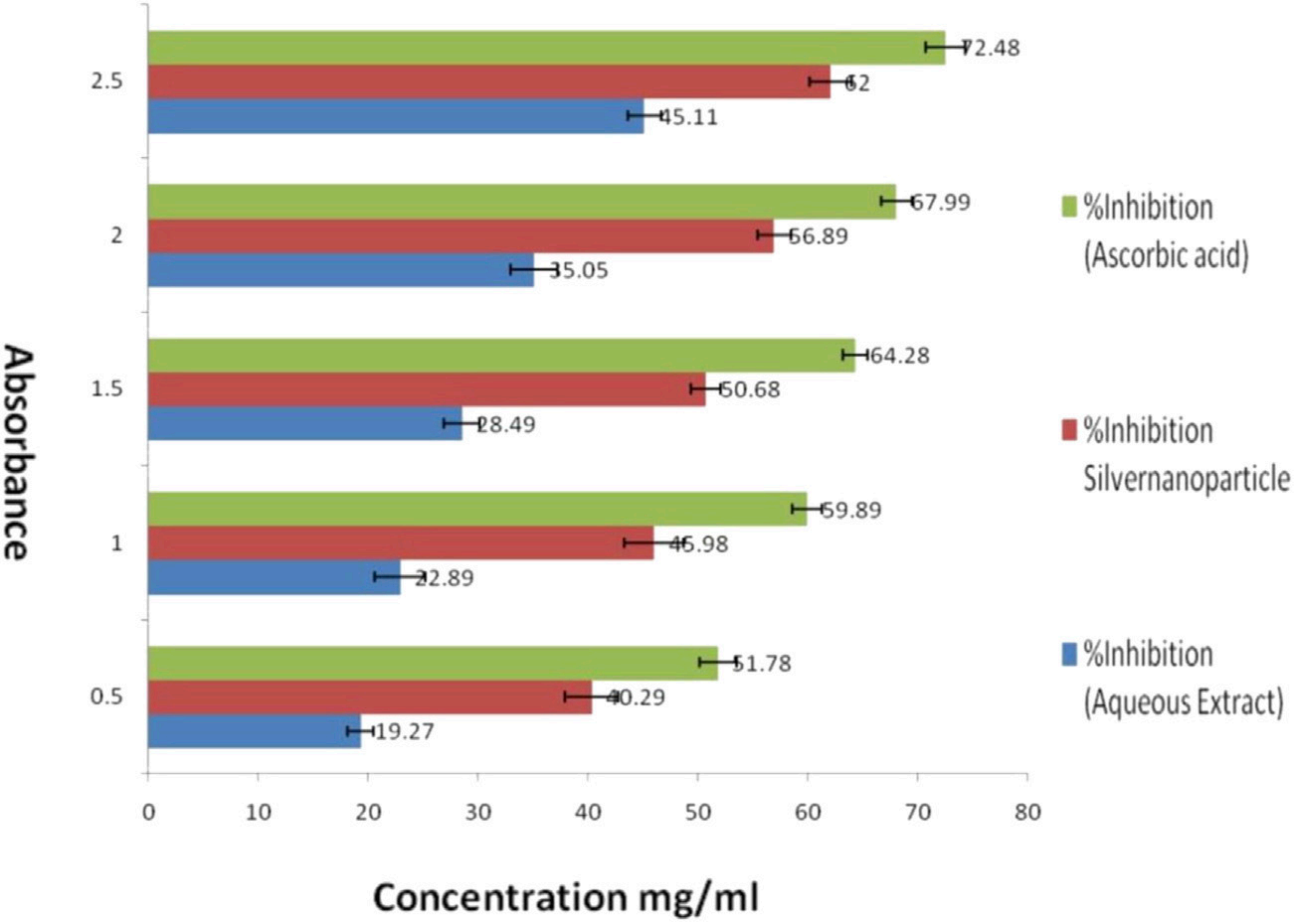
Evaluation of *in vitro* anti-oxidant activity of experimental Silver nanoparticles (AgNPs) by DPPH free radical scavenging assay against aqueous extract and ascorbic acid (standard).

An Examination of the antioxidant properties of optimized silver nanoparticles (AgNPs) produced from *Azadirachta indica* demonstrated promising findings for possible medicinal uses. When 2.5 mg/mL of aqueous extract was added, the enzyme activity was inhibited by 45.11%. However, using AgNPs resulted in a substantially greater inhibition rate of 62%. By contrast, ascorbic acid, a well-recognized antioxidant, attained an inhibition rate of 72.48%. The results suggest that AgNPs have a higher antioxidant capacity compared to the aqueous extract. The IC_50_ value, representing the concentration needed to block 50% of enzyme activity, was lower for AgNPs compared to the aqueous extract. This indicates that AgNPs have a greater ability to remove harmful free radicals and protect cells from oxidative stress. The study’s findings highlight the strong ability of the AgNPs to scavenge DPPH free radicals, hence strengthening their potential for use in the treatment of many disorders associated with oxidative stress. As shown in Table 2. The study’s findings highlight the strong ability of the AgNPs to scavenge DPPH free radicals, strengthening their potential for use in periodontal treatment as shown in Figure 8.

**TABLE 2:** *In vitro* anti-oxidant activity analysis of experimental AgNPs vs. aqueous extract through DPPH free radical scavenging assay; Ascorbic acid taken as standard.

Concentration mg/mL	%Inhibition (aqueous extract)	%Inhibition AgNPs	%Inhibition (Ascorbic acid)
0.5	19.27 ± 1.2	40.29 ± 2.4	51.78 ± 1.7
1	22.89 ± 2.3	45.98 ± 2.7	59.89 ± 1.3
1.5	28.49 ± 1.6	50.68 ± 1.4	64.28 ± 1.1
2	35.05 ± 2.1	56.89 ± 1.5	67.99 ± 1.4
2.5	45.11 ± 1.5	62.01 ± 1.9	72.48 ± 1.8

### 3.10 Molecular docking result

**FIGURE 9:**
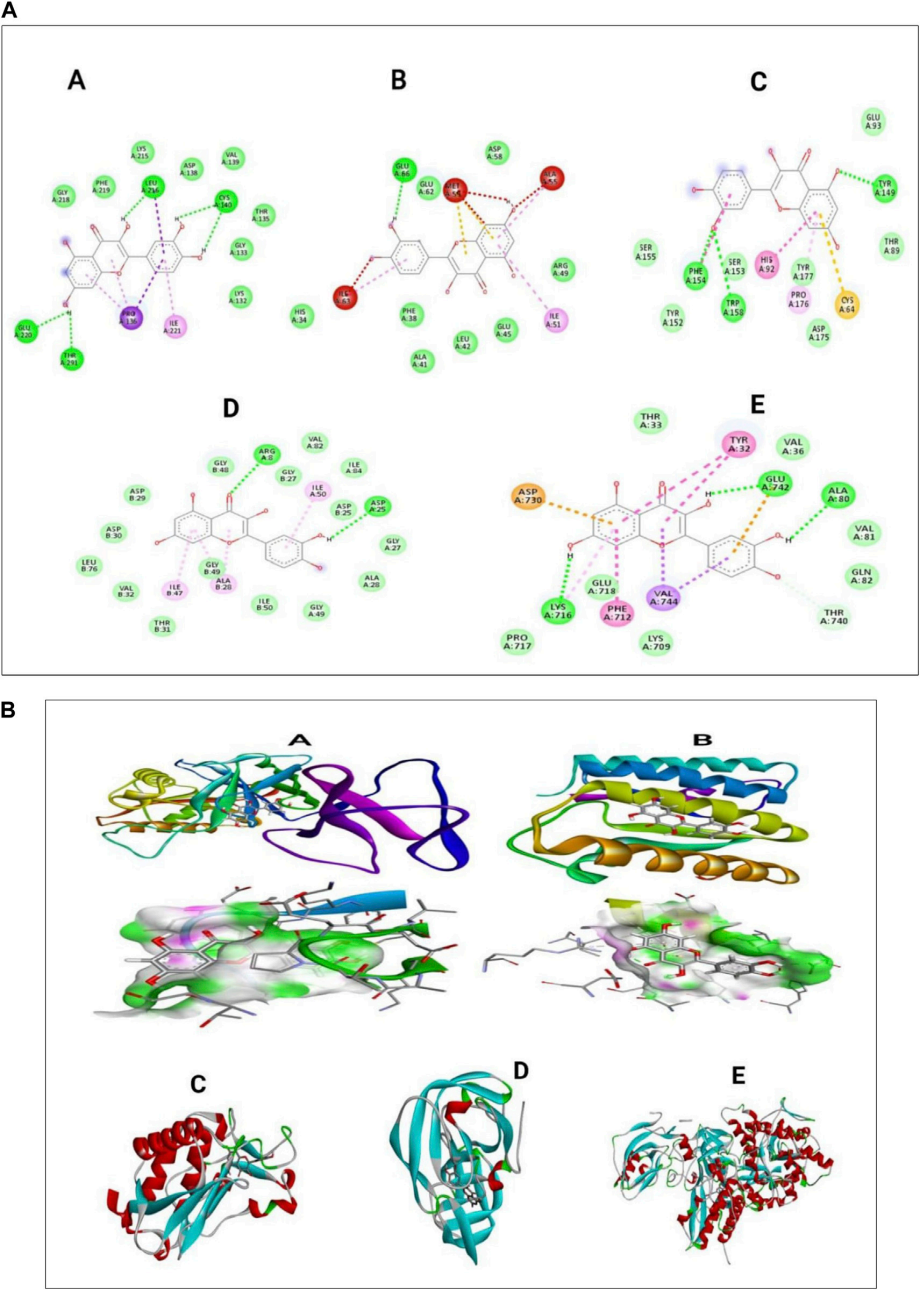
**(A)** In silico docking analysis of quercetin with five different selected proteins represented as two-dimensional images **(B)** Three-dimensional images of quercetin interacting with selected periodontal pathogenic proteins.

The presentation of the primary phytochemical was examined using *in silico* molecular docking simulation (Quercetin) on different virulence factor of selected periodontal causing bacteria’s. The findings demonstrated that quercetin interacted with distinct amino acids of the alkyl group at the active site of virulence factor of selected periodontal causing bacteria. These visualizations help to understand quercetin’s molecular interactions with enzymes, revealing its therapeutic potential. The docking algorithm was able to predict the Quercetin ligand pose with those mediators viz5Y1A and 3R15 binding energy score of-9.37151 kcal/mol and −9.04139 kcal/mol and amino acids in Collagenase, Protease, and Urease, resulting in binding energies of −7.3, −8.7, and −8.6 kcal/mol, respectively. The results indicate that a large negative score of the mediators viz5Y1A binding energy score of −9.37151 kcal/mol strongly binds with quercetin rather than other proteins (Wu et al., 2024; Keshari et al., 2020). In Figure 9A, the two-dimensional pictures illustrate how quercetin binds to specific sites on the enzymes, affecting their activity. The 3D visualizations in Figure 9B provide a more detailed look at the complex molecular interactions between quercetin and the enzymes, highlighting the specific structural changes that occur during the binding process (Aljelehawy et al., 2022).

### 3.11 *In-silico* Swiss-ADME analysis

The ADMET attribute of quercetin encompasses several characteristics, including physicochemical properties, lipophilicity, water solubility, pharmacokinetics, and drug-likeness. These attributes play a crucial role in determining the bioavailability and efficacy of quercetin as a potential drug candidate (Anwar et al., 2021).

As per Lipinski’s rule, the molecular mass is always less than 500 Da. This rule helps in predicting the likelihood of a compound being orally bioavailable. As per the result, quercetin has a molecular mass of 302.24 Da, which indicates it is a good conductor for the process. The number of H-bond acceptors is below 10, which is 7, which follows Lipinski rule 5. The molar reactivity is about 78.03, which is in the range of 40–130. This suggests that the compound has a moderate reactivity level that indicate a balance between the desired activity of the drug and its potential safety profile, making it potentially suitable for further drug development. The topological surface area can predict the solubility parameter of a compound, which is about (TPSA) 131.36 Å^2^. This value suggesting moderate absorption potential and the compound is unlikely to cross the BBB effectively, meaning it may not have central nervous system (CNS) effects. A TPSA under 140 Å^2^ is generally favorable for oral bioavailability, so this result suggests that the compound suitable for oral administration. The log P value, which is less than 5, is what determines a compound’s lipophilicity, and the average value is 1.22. The water solubility property of the compound shows that it is soluble in nature. The pharmacokinetic property shows high GI absorption; there is no blood-brain barrier permeation. The compound can be metabolized by the enzymes cytochrome P1A22, P2D6, P2D6, and P3A. The skin permeability log Kp is about 7.05 cm/s suggests that the compound has very high permeability through the skin. This is crucial for topical or transdermal drug delivery, as it indicates that the drug can effectively pass through the skin barrier and enter systemic circulation. Moderate reactivity and high skin permeability suggest that the compound could be effective in skin-based delivery methods.

The pharmacokinetics property shows the different parameters for designing a drug molecule and using the polymer to get more action. The drug-likeness property of the compound shows there is no violation of Lipinski, and the bioavailability score is about 0.55. A bioavailability score of 0.55 indicates that 55% of the orally administered compound reaches systemic circulation. This suggests that the compound has moderate oral absorption but may still be affected by factors like first-pass metabolism or poor permeability. The ADMET property of Quercetin was illustrated in the Table 3.

**TABLE 3:** Insight into ADMET property analysis of Quercetin using SWISS ADMET.

Physicochemical properties		Lipophilicity		Water solubility		Pharmaco-kinetics		Drug likeness	
Formula	C_15_H_10_O_7_	Log *P*_o/w_ (iLOGP)	1.63	Log *S* (ESOL)	−3.16	GI absorption	High	Lipinski	Yes; 0 violation
Molecular weight	302.24 g/mol	Log *P*_o/w_ (XLOGP3)	1.54	Solubility	2.11e-01 mg/mL; 6.98e-04 mol/L	BBB permeation	No	Ghose	Yes
Num. heavy atoms	22	Log *P*_o/w_ (WLOGP)	1.99	Class	Soluble	P-gp substrate	No	Veber	Yes
Num. arom. heavy atoms	16	Log *P*_o/w_ (MLOGP)	−0.56	Log *S* (Ali)	−3.91	CYP1A2 inhibitor	Yes	Egan	Yes
Fraction Csp3	0.00	Log *P*_o/w_ (SILICOS-IT)	1.54	Solubility	3.74e-02 mg/mL; 1.24e-04 mol/L	CYP2C19 inhibitor	No	Muegge	Yes
Num. rotatable bonds	1	Consensus Log *P*_o/w_	1.23	Class	Soluble	CYP2C9 inhibitor	No	Bioavailability Score	0.55
Num. H-bond acceptors	7			Log *S* (SILICOS-IT)	−3.24	CYP2D6 inhibitor	Yes		
Num. H-bond donors	5			Solubility	1.73e-01 mg/mL; 5.73e-04 mol/L	CYP3A4 inhibitor	Yes		
Molar Refractivity	78.03			Class	Soluble	Log *K*_p_ (skin permeation)	−7.05 cm/s		
TPSA	131.36 Å^2^								

**FIGURE 10:**
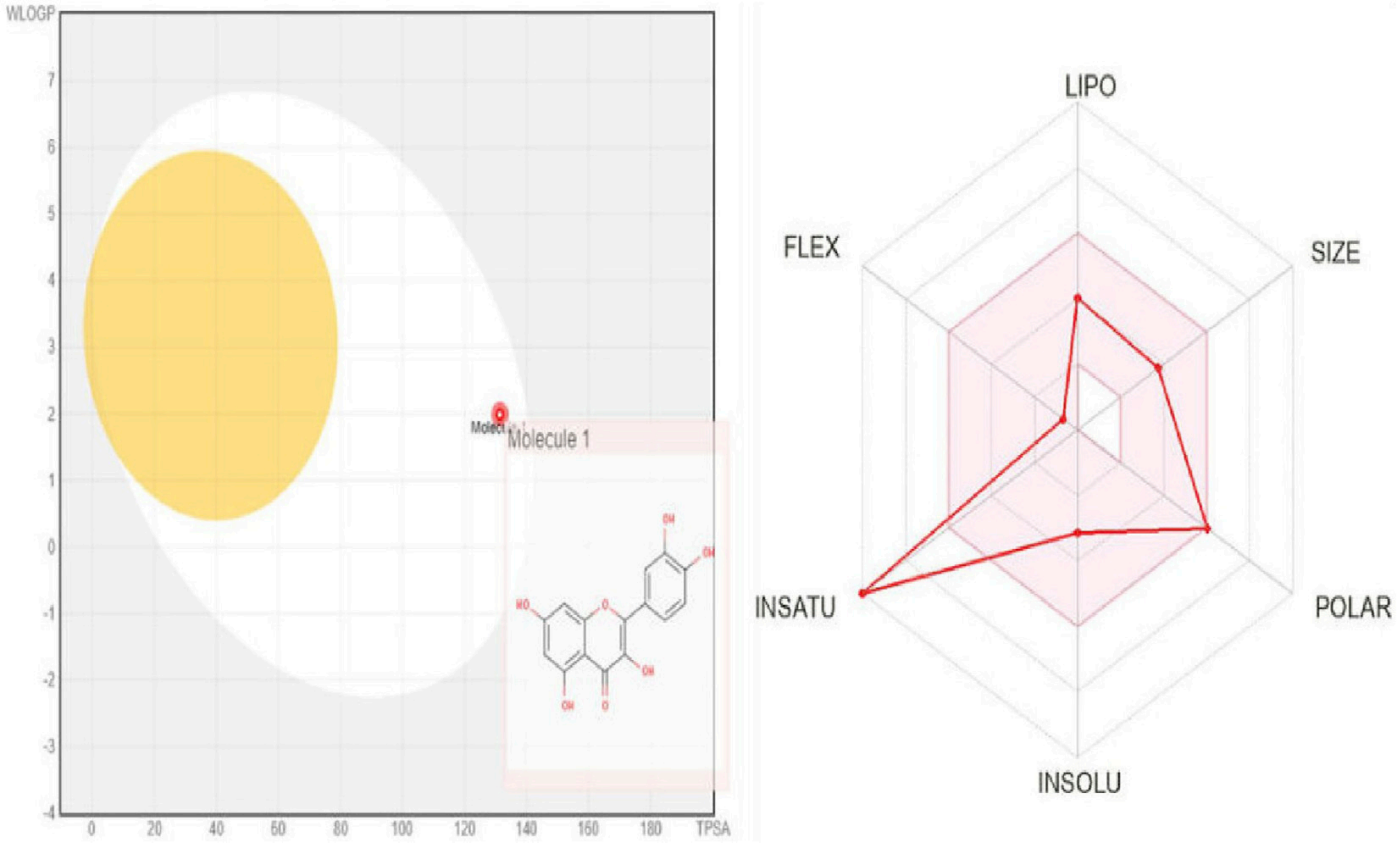
ADME analysis of quercetin using BOILED-Egg model.

The BOILED-Egg model simplifies the calculation of molecular polarity and lipophilicity. According to the findings of the BOILED-Egg model, the molecules would appear in the white section of the egg, which would indicate that they were absorbed by the gastrointestinal tract. The BOILED-Egg model provides a visual representation of where the molecule is likely to be absorbed in the body, guiding further research and development efforts. Overall, the compound appears to have favorable properties for drug development, with high solubility, absorption, and metabolism rates. The skin permeability and pharmacokinetics data suggest that the compound could be effective when administered orally or through the skin. Additionally, the lack of blood-brain barrier permeation indicates that the compound may have a lower risk of causing central nervous system side effects. The adherence to Lipinski’s rule of five and favorable bioavailability score further support the potential of this compound as a drug candidate [69]. The BOILED-Egg model of Quercetin was illustrated (Figure 10).

## 4 Conclusion

One easy, green, and inexpensive way to make AgNPs is to use the extract from the leaves of the *Azadirachta indica* plant. The *Azadirachta indica* leaves contain phytochemicals, including flavonoid (Quercetin), which function as both reducing and capping agents throughout the synthesis process. The silver nanoparticles (AgNPs) show strong antibacterial efficacy against pathogenic gram-negative strains. The ability of AgNPs to break down bacterial cell membranes, stop metabolic processes, and make reactive oxygen species makes them antibacterial. The *Azadirachta indica* derived AgNPs not only possess antibacterial activities but also exhibit substantial antioxidant activity. The *Azadirachta indica* leaves include phytochemicals that enhance the ability of the AgNPs to neutralize free radicals and reduce them. Their size was determined to be 86 nm, with a potential of −26.9 mV. Furthermore, the SAED, FTIR, AFM, and TEM analyses demonstrated advantageous morphological properties of the silver nanoparticles. The promising results from *in silico* docking and ADME analyses demonstrated that Quercetin exhibits strong binding affinity to the target protein, which is critically involved in bacterial growth and biofilm formation on teeth. Additionally, ADME analysis indicated high skin permeability, supporting the potential of a synergistic combination of Quercetin and silver nanoparticles for effective topical application in periodontal therapy. The detailed study of the synthesized AgNPs gives us important information about their structure and function, which supports the idea that they could be used to treat gum disease. The study indicates that the synthesized AgNPs have acceptable pharmacokinetic features and low toxicity, making them appropriate for biomedical applications, particularly periodontal usage.

## 5 Future scope

Anticipating the future, the potential of this study is quite promising. Continued improvement of the synthesis process and optimization approaches may result in the powerful formulations that have improved medicinal benefits. Furthermore, exploring the possible synergistic impacts with other therapeutic agents might open up novel approaches for periodontal disorders. Additionally, continued research into the effectiveness and safety of these novel treatments will be crucial in advancing the field of periodontics.

## Data Availability

The original contributions presented in the study are included in the article/Supplementary Material, further inquiries can be directed to the corresponding authors.

## Abbreviations

AgNPs, Silver nanoparticle; FTIR, Fourier transmission infrared spectroscopy; TEM, Transmission electron microscopy; DPPH, 2-Phenyl-1-Picrylhydrazyl; SAED, Selected area electron diffraction, MIC, Minimum Inhibitory Concentration; TPSA, Topological surface area.

## References

[B1] Abdel-Aty A. M. Barakat A. Z. Bassuiny R. I. Mohamed S. A. (2023). Statistical optimization, characterization, antioxidant and antibacterial properties of silver nanoparticle biosynthesized by saw palmetto seed phenolic extract. Sci. Rep. 13 (1), 15605. 10.1038/s41598-023-42675-0 37731031 PMC10511706

[B2] Adaramola F. Adewole S. Adewole O. (2023). Assessment of phytochemicals, antioxidant and antimicrobial activities of aqueous ethanol extract and fractions of Azadirachta indica stem bark. Int. J. Sci. Glob. Sustain. 9 (1), 13. 10.57233/ijsgs.v9i1.401

[B3] Aljelehawy Q. Mal Allah O. R. Sourazur G. (2022). Physicochemical properties, medicinal chemistry, toxicity, and absorption of quercetin and its interaction with spike glycoprotein of SARS-CoV-2: molecular docking. Nano Micro Biosyst. 1 (1), 32–39. 10.22034/nmbj.2022.163207

[B4] Amananti W. Riyantal A. B. Kusnadi K. Aledresi K. A. M. S. (2022). Green synthesis and antibacterial activity of silver nanoparticles using turi (Sesbania grandiflora lour) leaf extract. EKSAKTA Berk. Ilm. Bid. MIPA 23 (04), 255–265. 10.24036/eksakta/vol23-iss04/337

[B5] Anwar S. S. Al-Shmgani H. S. Tawfeeq A. T. Sulaiman G. M. Al-Mousawi Y. H. (2021). *In silico* analysis of quercetin as potential anti-cancer agents. Mater. Today Proc. 42, 2521–2526. 10.1016/j.matpr.2020.12.573

[B6] Bajpai R. Roy S. Verma S. (2022). Microwave-assisted solid-state synthesis of dichalcogenide nanostructures for electrocatalytic hydrogen evolution. ACS Appl. Nano Mater. 5 (6), 8511–8525. 10.1021/acsanm.2c01908

[B7] Chavan R. R. Bhutkar M. A. Bhinge S. D. (2023). Design, synthesis, and optimization of silver nanoparticles using an Artocarpus heterophyllus Lam. leaf extract and its antibacterial application. Nano Biomed. Eng. 15 (3), 239–252. 10.26599/nbe.2023.9290011

[B8] Chinnasamy G. Chandrasekharan S. Koh T. W. Bhatnagar S. (2021). Synthesis, characterization, antibacterial and wound healing efficacy of silver nanoparticles from Azadirachta indica. Front. Microbiol. 12, 611560. 10.3389/fmicb.2021.611560 33679635 PMC7932996

[B9] Dashora A. Rathore K. Raj S. Sharma K. (2022). Synthesis of silver nanoparticles employing Polyalthia longifolia leaf extract and their *in vitro* antifungal activity against phytopathogen. Biochem. Biophysics Rep. 31, 101320. 10.1016/j.bbrep.2022.101320 PMC9398913 36032398

[B10] Donga S. Chanda S. (2021). Facile green synthesis of silver nanoparticles using Mangifera indica seed aqueous extract and its antimicrobial, antioxidant and cytotoxic potential (3-in-1 system). Artif. Cells, Nanomedicine, Biotechnol. 49 (1), 292–302. 10.1080/21691401.2021.1899193 33733973

[B11] Dubey P. Mittal N. (2020). Periodontal diseases-a brief review. Int. J. Oral Heal Dent. 6 (3), 177–187. 10.18231/j.ijohd.2020.038

[B12] Dutt Y. Pandey R. P. Dutt M. Gupta A. Vibhuti A. Raj V. S. (2023). Silver nanoparticles phytofabricated through Azadirachta indica: anticancer, apoptotic, and wound-healing properties. Antibiotics 12 (1), 121. 10.3390/antibiotics12010121 36671322 PMC9855199

[B13] Gidiagba J. O. Daraojimba C. Ofonagoro K. A. Eyo-Udo N. L. Egbokhaebho B. A. Ogunjobi O. A. (2023). Economic impacts and innovations in materials science: a holistic exploration of nanotechnology and advanced materials. Eng. Sci. and Technol. J. 4 (3), 84–100. 10.51594/estj.v4i3.553

[B14] Gonfa Y. H. Gelagle A. A. Hailegnaw B. Kabeto S. A. Workeneh G. A. Tessema F. B. (2023). Optimization, characterization, and biological applications of silver nanoparticles synthesized using essential oil of aerial part of Laggera tomentosa. Sustainability 15 (1), 797. 10.3390/su15010797

[B15] Gontijo L. A. P. Raphael E. Ferrari D. P. S. Ferrari J. L. Lyon J. P. Schiavon M. A. (2020). pH effect on the synthesis of different size silver nanoparticles evaluated by DLS and their size-dependent antimicrobial activity. Matéria (Rio de Janeiro) 25 (04), e–12845. 10.1590/s1517-707620200004.1145

[B16] Grizzo A. Dos Santos D. M. da Costa V. P. Lopes R. G. Inada N. M. Correa D. S. (2023). Multifunctional bilayer membranes composed of poly (lactic acid), beta-chitin whiskers and silver nanoparticles for wound dressing applications. Int. J. Biol. Macromol. 251, 126314. 10.1016/j.ijbiomac.2023.126314 37586628

[B17] Gupta D. Boora A. Thakur A. Gupta T. K. (2023). Green and sustainable synthesis of nanomaterials: recent advancements and limitations. Environ. Res. 231, 116316. 10.1016/j.envres.2023.116316 37270084

[B18] Habeeb Rahuman H. B. Dhandapani R. Narayanan S. Palanivel V. Paramasivam R. Subbarayalu R. (2022). Medicinal plants mediated the green synthesis of silver nanoparticles and their biomedical applications. IET nanobiotechnology 16 (4), 115–144. 10.1049/nbt2.12078 35426251 PMC9114445

[B19] Halilu E. M. Ngweh V. A. Airemwen C. O. (2023). Green synthesis of silver nanoparticles from Parinari curatellifolia methanol stem bark extract and evaluation of antioxidant and antimicrobial activities. Trop. J. Nat. Prod. Res. 7 (3). 10.26538/tjnpr/v7i3.5

[B20] Hameed A. Rehman T. U. Rehan Z. A. Noreen R. Iqbal S. Batool S. (2022). Development of polymeric nanofibers blended with extract of neem (Azadirachta indica), for potential biomedical applications. Front. Mater. 9, 1042304. 10.3389/fmats.2022.1042304

[B21] Harish V. Ansari M. M. Tewari D. Yadav A. B. Sharma N. Bawarig S. (2023). Cutting-edge advances in tailoring size, shape, and functionality of nanoparticles and nanostructures: a review. J. Taiwan Inst. Chem. Eng. 149, 105010. 10.1016/j.jtice.2023.105010

[B22] Hasan M. M. Khan Z. Chowdhury M. S. Khan M. A. Moni M. A. Rahman M. H. (2022). *In silico* molecular docking and ADME/T analysis of Quercetin compound with its evaluation of broad-spectrum therapeutic potential against particular diseases. Inf. Med. Unlocked 29, 100894. 10.1016/j.imu.2022.100894

[B23] Hashemi Z. Shirzadi-Ahodashti M. Mortazavi-Derazkola S. Ebrahimzadeh M. A. (2022). Sustainable biosynthesis of metallic silver nanoparticles using barberry phenolic extract: optimization and evaluation of photocatalytic, *in vitro* cytotoxicity, and antibacterial activities against multidrug-resistant bacteria. Inorg. Chem. Commun. 139, 109320. 10.1016/j.inoche.2022.109320

[B68] Hedayatipanah M. Gholami L. Farmany A. Alikhani M. Y. Hooshyarfard A. Hashemiyan F. S. (2024). Green synthesis of silver nanoparticles and evaluation of their effects on the Porphyromonas gingivalis bacterial biofilm formation. Clin. Exp. Dent. Res. 10 (3), e887. 10.1002/cre2.887 38798089 PMC11128748

[B24] Hernández-Venegas P. A. Martínez-Martínez R. E. Zaragoza-Contreras E. A. Domínguez-Pérez R. A. Reyes-López S. Y. Donohue-Cornejo A. (2023). Bactericidal activity of silver nanoparticles on oral biofilms related to patients with and without periodontal disease. J. Funct. Biomaterials 14 (6), 311. 10.3390/jfb14060311 PMC10299358 37367275

[B25] Hossain N. Islam M. A. Ahmed M. M. S. Chowdhury M. A. Mobarak M. H. Rahman M. M. (2024). Advances and significances of titaniumin dental implant applications. Results Chem. 7, 101394. 10.1016/j.rechem.2024.101394

[B26] Hu X. Zuo D. Cheng S. Chen S. Liu Y. Bao W. (2023). Ultrafast materials synthesis and manufacturing techniques for emerging energy and environmental applications. Chem. Soc. Rev. 52 (3), 1103–1128. 10.1039/d2cs00322h 36651148

[B27] Huq M. A. Ashrafudoulla M. Rahman M. M. Balusamy S. R. Akter S. (2022). Green synthesis and potential antibacterial applications of bioactive silver nanoparticles: a review. Polymers 14 (4), 742. 10.3390/polym14040742 35215655 PMC8879957

[B28] Ibrahim S. Ahmad Z. Manzoor M. Z. Mujahid M. Faheem Z. Adnan A. (2021). Optimization for biogenic microbial synthesis of silver nanoparticles through response surface methodology, characterization, their antimicrobial, antioxidant, and catalytic potential. Sci. Rep. 11 (1), 770. 10.1038/s41598-020-80805-0 33436966 PMC7804320

[B29] Ijaz I. Bukhari A. Gilani E. Nazir A. Zain H. Saeed R. (2022). Green synthesis of silver nanoparticles using different plants parts and biological organisms, characterization and antibacterial activity. Environ. Nanotechnol. Monit. and Manag. 18, 100704. 10.1016/j.enmm.2022.100704

[B30] Imchen P. Ziekhrü M. Zhimomi B. K. Phucho T. (2022). Biosynthesis of silver nanoparticles using the extract of Alpinia galanga rhizome and Rhus semialata fruit and their antibacterial activity. Inorg. Chem. Commun. 142, 109599. 10.1016/j.inoche.2022.109599

[B31] Karan T. Gonulalan Z. Erenler R. Kolemen U. Eminagaoglu O. (2024). Green synthesis of silver nanoparticles using Sambucus ebulus leaves extract: characterization, quantitative analysis of bioactive molecules, antioxidant and antibacterial activities. J. Mol. Struct. 1296, 136836. 10.1016/j.molstruc.2023.136836

[B32] Kemala P. Idroes R. Khairan K. Ramli M. Jalil Z. Idroes G. M. (2022). Green synthesis and antimicrobial activities of silver nanoparticles using Calotropis gigantea from Ie Seu-Um Geothermal area, Aceh Province, Indonesia. Molecules 27 (16), 5310. 10.3390/molecules27165310 36014547 PMC9415655

[B33] Keshari A. K. Srivastava R. Singh P. Yadav V. B. Nath G. (2020). Antioxidant and antibacterial activity of silver nanoparticles synthesized by Cestrum nocturnum. J. Ayurveda Integr. Med. 11 (1), 37–44. 10.1016/j.jaim.2017.11.003 30120058 PMC7125370

[B34] Khan Y. Sadia H. Ali Shah S. Z. Khan M. N. Shah A. A. Ullah N. (2022). Classification, synthetic, and characterization approaches to nanoparticles, and their applications in various fields of nanotechnology: a review. Catalysts 12 (11), 1386. 10.3390/catal12111386

[B35] Kumar Y. Paswan K. K. Nayan K. Pandurangappa G. Dwivedi D. Sangwai J. S. (2023). “Introduction to functional materials: synthesis, properties, environmental sustainability, and general applications,” in Functional materials for the oil and gas industry (London: CRC Press), 1–22.

[B36] Liaqat N. Jahan N. Anwar T. Qureshi H. (2022). Green synthesized silver nanoparticles: optimization, characterization, antimicrobial activity, and cytotoxicity study by hemolysis assay. Front. Chem. 10, 952006. 10.3389/fchem.2022.952006 36105303 PMC9465387

[B37] Lomelí-Rosales D. A. Zamudio-Ojeda A. Reyes-Maldonado O. K. López-Reyes M. E. Basulto-Padilla G. C. Lopez-Naranjo E. J. (2022). Green synthesis of gold and silver nanoparticles using leaf extract of Capsicum chinense plant. Molecules 27 (5), 1692. 10.3390/molecules27051692 35268794 PMC8911899

[B38] Malandrakis A. A. Kavroulakis N. Chrysikopoulos C. V. (2022). Metal nanoparticles against fungicide resistance: alternatives or partners? Pest Manag. Sci. 78 (10), 3953–3956. 10.1002/ps.7014 35620887

[B39] Mallineni S. K. Sakhamuri S. Kotha S. L. AlAsmari A. R. G. M. AlJefri G. H. Almotawah F. N. (2023). Silver nanoparticles in dental applications: a descriptive review. Bioengineering 10 (3), 327. 10.3390/bioengineering10030327 36978718 PMC10044905

[B40] Moges A. Goud V. V. (2022). Optimization, characterization, and evaluation of antioxidant and antibacterial activities of silver nanoparticles synthesized from Hippophae salicifolia D. Don. Inorg. Chem. Commun. 146, 110086. 10.1016/j.inoche.2022.110086

[B41] Muraro P. C. L. Pinheiro L. D. S. M. Chuy G. Vizzotto B. S. Pavoski G. Espinosa D. C. R. (2022). Silver nanoparticles from residual biomass: biosynthesis, characterization and antimicrobial activity. J. Biotechnol. 343, 47–51. 10.1016/j.jbiotec.2021.11.003 34826535

[B42] Nelagadarnahalli H. J. Jacob G. K. Prakash D. Iska R. R. Iska V. B. R. Ameen F. (2023). Optimization and fabrication of silver nanoparticles to assess the beneficial biological effects besides the inhibition of pathogenic microbes and their biofilms. Inorg. Chem. Commun. 156, 111140. 10.1016/j.inoche.2023.111140

[B43] Nesappan T. Subramani A. (2023). Preparation of implant-prosthetic disinfectant solution by chitosan, green synthesized silver nanoparticles using cymbopogon citratus, azadirachta indica, melaleuca alternifolia and evaluation of its antimicrobial properties-an invitro study. J. Pharm. Negat. Results 14 (2). 10.47750/pnr.2023.14.02.241

[B44] Ortega F. Versino F. López O. V. García M. A. (2022). Biobased composites from agro-industrial wastes and by-products. Emergent Mater. 5 (3), 873–921. 10.1007/s42247-021-00319-x 34849454 PMC8614084

[B45] Osherov A. Prasad R. Chrzanowski W. New E. J. Brazaca L. Sadik O. (2023). Responsible nanotechnology for a sustainable future. One Earth 6 (7), 763–766. 10.1016/j.oneear.2023.06.010

[B46] Owaid M. N. Rabeea M. A. Aziz A. A. Jameel M. S. Dheyab M. A. (2021). “Mycogenic fabrication of silver nanoparticles using Picoa,” in Pezizales, characterization and their antifungal activity. 10.1016/j.enmm.2021.100612

[B47] Ozdal M. Gurkok S. (2022). Recent advances in nanoparticles as antibacterial agent. ADMET DMPK 10 (2), 115–129. 10.5599/admet.1172 35350114 PMC8957245

[B48] Prakash V. Akhtar S. Kumar J. Mishra S. K. Pandey R. R. (2022). “Azadirachta indica: the multifaceted and versatile tree,” in Current trends in medicinal chemistry.

[B49] Salem H. F. Nafady M. M. Ewees M. G. E. D. Hassan H. Khallaf R. A. (2022). Rosuvastatin calcium-based novel nanocubic vesicles capped with silver nanoparticles-loaded hydrogel for wound healing management: optimization employing Box–Behnken design: *in vitro* and *in vivo* assessment. J. Liposome Res. 32 (1), 45–61. 10.1080/08982104.2020.1867166 33353435

[B50] Satapathy B. S. Mishra A. Biswal S. K. Pattnaik S. Parida R. Biswal B. (2024). Encapsulation of Alpinia leaf essential oil in nanophytosome-embedded gel as novel strategy to treat periodontal infections: evaluation of antimicrobial effectiveness, pharmacokinetic, *in vitro*-*ex vivo* correlation and *in silico* studies. J. Microencapsul. 41, 327–344. 10.1080/02652048.2024.2354234 38829223

[B51] Sedghi L. DiMassa V. Harrington A. Lynch S. V. Kapila Y. L. (2021). The oral microbiome: role of key organisms and complex networks in oral health and disease. Periodontology 87 (1), 107–131. 10.1111/prd.12393 PMC8457218 34463991

[B52] Shareef M. A. Sirisha K. Sayeed I. B. Khan I. Ganapathi T. Akbar S. (2019). Synthesis of new triazole fused imidazo[2,1-b]thiazole hybrids with emphasis on *Staphylococcus aureus* virulence factors. Bioorg. and Med. Chem. Lett. 29 (19), 126621. 10.1016/j.bmcl.2019.08.025 31431360

[B53] Sharifi-Rad M. Elshafie H. S. Pohl P. (2024). Green synthesis of silver nanoparticles (AgNPs) by Lallemantia royleana leaf extract: their bio-pharmaceutical and catalytic properties. J. Photochem. Photobiol. A Chem. 448, 115318. 10.1016/j.jphotochem.2023.115318

[B54] Sharma N. K. Vishwakarma J. Rai S. Alomar T. S. AlMasoud N. Bhattarai A. (2022). Green route synthesis and characterization techniques of silver nanoparticles and their biological adeptness. ACS omega 7 (31), 27004–27020. 10.1021/acsomega.2c01400 35967040 PMC9366950

[B55] Shirmohammadi A. Maleki Dizaj S. Sharifi S. Fattahi S. Negahdari R. Ghavimi M. A. (2023). Promising antimicrobial action of sustained released curcumin-loaded silica nanoparticles against clinically isolated Porphyromonas gingivalis. Diseases 11 (1), 48. 10.3390/diseases11010048 36975597 PMC10047251

[B56] Singh A. Gaud B. Jaybhaye S. (2020). Optimization of synthesis parameters of silver nanoparticles and its antimicrobial activity. Mater. Sci. Energy Technol. 3, 232–236. 10.1016/j.mset.2019.08.004

[B57] Singh P. Mijakovic I. (2022a). Green synthesis and antibacterial applications of gold and silver nanoparticles from Ligustrum vulgare berries. Sci. Rep. 12 (1), 7902. 10.1038/s41598-022-11811-7 35551489 PMC9098411

[B58] Singh P. Mijakovic I. (2022b). Strong antimicrobial activity of silver nanoparticles obtained by the green synthesis in Viridibacillus sp. extracts. Front. Microbiol. 13, 820048. 10.3389/fmicb.2022.820048 35250934 PMC8888960

[B59] Vijayaram S. Razafindralambo H. Sun Y. Z. Vasantharaj S. Ghafarifarsani H. Hoseinifar S. H. (2024). Applications of green synthesized metal nanoparticles—a review. Biol. Trace Elem. Res. 202 (1), 360–386. 10.1007/s12011-023-03645-9 37046039 PMC10097525

[B60] Wade W. G. (2021). Resilience of the oral microbiome. Periodontology 86 (1), 113–122. 10.1111/prd.12365 33690989

[B61] Wasilewska A. Klekotka U. Zambrzycka M. Zambrowski G. Święcicka I. Kalska-Szostko B. (2023). Physico-chemical properties and antimicrobial activity of silver nanoparticles fabricated by green synthesis. Food Chem. 400, 133960. 10.1016/j.foodchem.2022.133960 36063680

[B62] Wu T. Fu Y. Guo S. Shi Y. Zhang Y. Fan Z. (2024). Self assembly multifunctional DNA tetrahedron for efficient elimination of antibiotic resistant bacteria. Aggregate 5 (1), e402. 10.1002/agt2.402

[B63] Xu Q. Cai H. Li W. Wu M. Wu Y. Gong X. (2022). Carbon dot/inorganic nanomaterial composites. J. Mater. Chem. A 10 (28), 14709–14731. 10.1039/d2ta02628g

[B64] Yang B. Yang H. Liang J. Chen J. Wang C. Wang Y. (2024). A review on the screening methods for the discovery of natural antimicrobial peptides. J. Pharm. Analysis, 101046. 10.1016/j.jpha.2024.101046 PMC11780100 39885972

[B65] Yun Z. Qin D. Wei F. Xiaobing L. (2022). Application of antibacterial nanoparticles in orthodontic materials. Nanotechnol. Rev. 11 (1), 2433–2450. 10.1515/ntrev-2022-0137

[B66] Zhang L. Shi H. Tan X. Jiang Z. Wang P. Qin J. (2022b). Ten-gram-scale mechanochemical synthesis of ternary lanthanum coordination polymers for antibacterial and antitumor activities. Front. Chem. 10, 898324. 10.3389/fchem.2022.898324 35774860 PMC9237552

[B67] Zhang S. Lin L. Huang X. Lu Y. G. Zheng D. L. Feng Y. (2022a). Antimicrobial properties of metal nanoparticles and their oxide materials and their applications in oral biology. J. Nanomater. 2022 (1), 2063265. 10.1155/2022/2063265

