# Supplementary figures and images for: Sustainable synthesis of silver nanoparticles from Azadirachta indica: antimicrobial, antioxidant and in silico analysis for periodontal treatment
Source: Front Chem. 2024 Oct 15;12:1489253. doi: 10.3389/fchem.2024.1489253 (PMC11518748; doi:10.3389/fchem.2024.1489253)

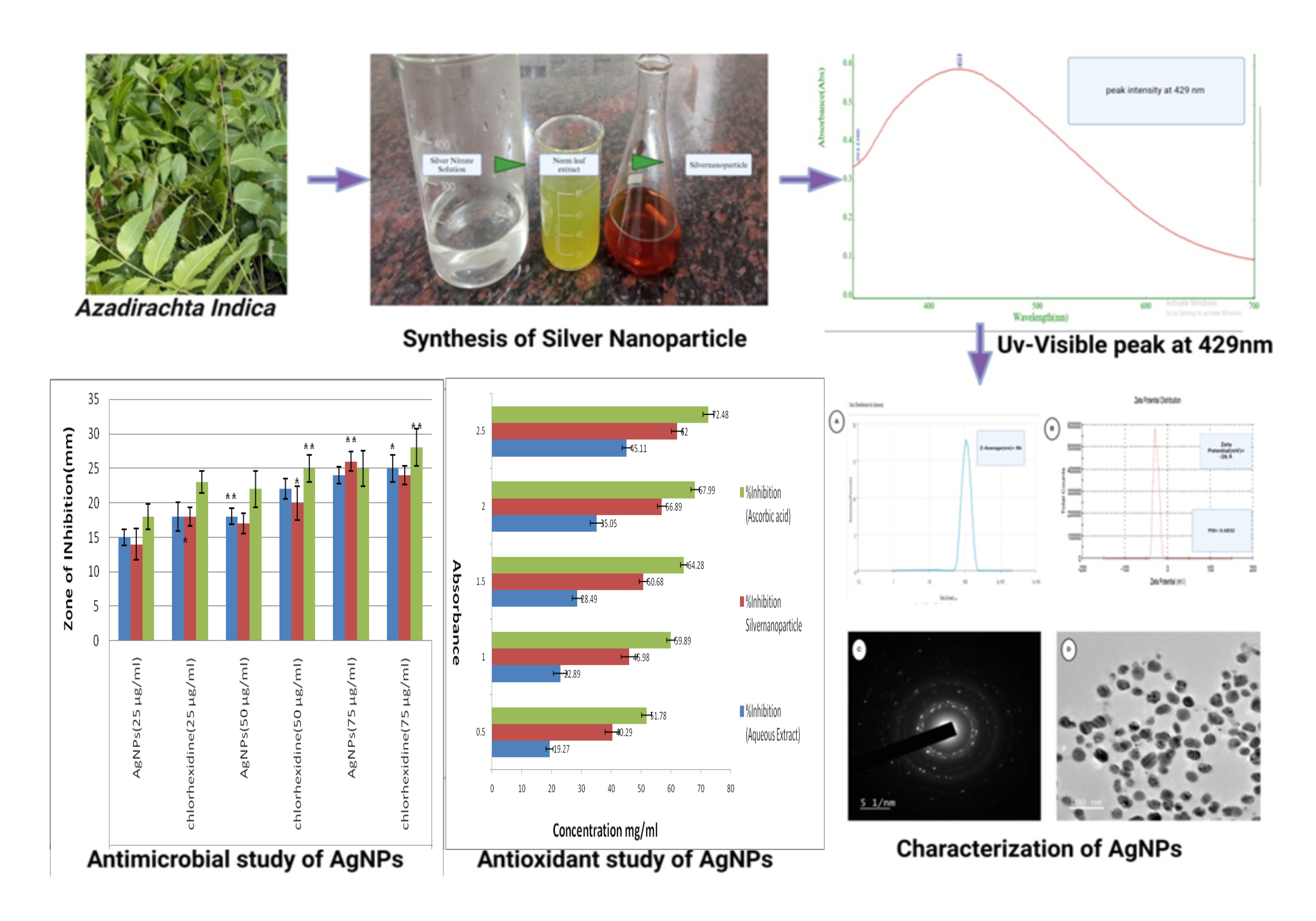

Supplement: Supplementary file 1 [file Image1.JPEG]
